# Chemokine receptors coordinately regulate macrophage dynamics and mammary gland development

**DOI:** 10.1242/dev.187815

**Published:** 2020-06-17

**Authors:** Gillian J. Wilson, Ayumi Fukuoka, Samantha R. Love, Jiwon Kim, Marieke Pingen, Alan J. Hayes, Gerard J. Graham

**Affiliations:** 1 Chemokine Research Group, Institute of Infection, Immunity and Inflammation, University of Glasgow, 120 University Place, Glasgow G12 8TA, UK; 2 Department of Physiology, University of Toronto, Medical Sciences Building, 1 King's College Circle, Toronto, ON M5S 1A8, Canada

**Keywords:** Chemokine, Macrophage, Mammary, Branching, Puberty, CCR1

## Abstract

Macrophages are key regulators of developmental processes, including those involved in mammary gland development. We have previously demonstrated that the atypical chemokine receptor ACKR2 contributes to the control of ductal epithelial branching in the developing mammary gland by regulating macrophage dynamics. ACKR2 is a chemokine-scavenging receptor that mediates its effects through collaboration with inflammatory chemokine receptors (iCCRs). Here, we reveal reciprocal regulation of branching morphogenesis in the mammary gland, whereby stromal ACKR2 modulates levels of the shared ligand CCL7 to control the movement of a key population of CCR1-expressing macrophages to the ductal epithelium. In addition, oestrogen, which is essential for ductal elongation during puberty, upregulates CCR1 expression on macrophages. The age at which girls develop breasts is decreasing, which raises the risk of diseases including breast cancer. This study presents a previously unknown mechanism controlling the rate of mammary gland development during puberty and highlights potential therapeutic targets.

## Abstract (precis)

**Summary:** The timing of pubertal mammary gland development is determined by availability of the chemokine CCL7, balanced by the scavenging receptor ACKR2 and the macrophage receptor CCR1.

## INTRODUCTION

Breast development (thelarche) is the first visible sign of puberty in females, and typically occurs between the ages of 8 and 13 (Merke and Cutler, 1996). Globally, the age of pubertal onset is falling (de Muinck Keizer-Schrama and Mul, 2001). Early puberty is associated with an increased risk of disease in later life, including type II diabetes, heart disease and cancer (Day et al., 2015). Importantly, girls who develop breasts before the age of 10 are 20% more likely to develop breast cancer (Bodicoat et al., 2014). Therefore, understanding the molecular and cellular mechanisms underlying breast development is of key importance.

The mammary gland develops through branching morphogenesis, giving rise to ductal epithelial networks. In the mouse, this process begins at around 3 weeks (Richert et al., 2000), when highly proliferative structures known as terminal end buds (TEBs) form at the end of epithelial ducts and drive network formation. Supporting this process is a stromal population containing fibroblasts, extracellular matrix (ECM), adipocytes and immune cells (Wiseman and Werb, 2002). Prominent among the stromal immune cells are macrophages, which are found throughout the gland and surrounding TEBs. Macrophages have been implicated in numerous developmental processes (Wynn et al., 2013), and mammary gland development is severely impaired in macrophage-deficient mice with altered TEB formation, ductal elongation during puberty and lobuloalveoli development in pregnancy (Pollard and Hennighausen, 1994; Gouon-Evans et al., 2000). Overall, these studies indicate a key role for macrophages in the regulation of ductal branching in the developing mammary gland.

Macrophages are recruited in a dynamic manner into the mammary gland throughout development (Coussens and Pollard, 2011). The molecular mechanisms regulating the intra-gland movement of macrophages, as they migrate to terminal end buds to mediate their developmental effects, are not currently understood and insights into these mechanisms will enhance our overall understanding of how macrophages control mammary gland development. Chemokines, which make up a family of proteins characterised by a conserved cysteine motif, are important *in vivo* regulators of macrophage intra-tissue dynamics. The chemokine family is subdivided into CC, CXC, XC and CX3C subfamilies according to the cysteine distribution, and chemokines act through G-protein-coupled receptors to mediate leukocyte migration (Nibbs and Graham, 2013). Within tissues, chemokine distribution and gradients can be regulated by members of the atypical chemokine receptor (ACKR) family, which are 7-transmembrane spanning receptors that lack classical signalling responses to ligands and are typically stromally expressed (Nibbs and Graham, 2013). Therefore, together, signalling chemokine receptors and ACKRs regulate intra-tissue chemokine function and coordinate leukocyte migration.

**Fig. 1.**
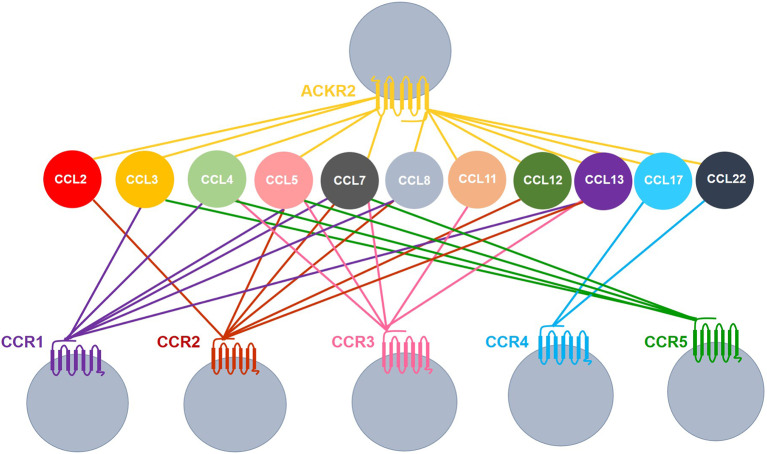
**ACKR2 shares ligands with inflammatory chemokine receptors.** Coloured lines indicate receptor-ligand interactions. Image compiled using data from Bachelerie et al. (2014) and Nibbs and Graham (2013).

We have a long-standing interest in one of the atypical chemokine receptors, ACKR2. ACKR2 scavenges and degrades inflammatory CC chemokines, thereby regulating their intra-tissue concentration and spatial distribution (Nibbs and Graham, 2013). Accordingly, it is a key player in the resolution of the inflammatory response with implications for autoimmunity and cancer (Nibbs et al., 2007; Di Liberto et al., 2008; Shams et al., 2017). We have previously demonstrated a role for ACKR2 in regulating branching morphogenesis in the developing lymphatic system via control of macrophage dynamics around developing vessels (Lee et al., 2014). More recently, we have shown that ACKR2 also regulates branching morphogenesis in the mammary gland and *Ackr2*^−/−^ mice display precocious mammary gland development. In essence, ACKR2 deficiency results in increased levels of monocyte- and macrophage-attracting chemokines in the developing mammary gland; this is associated with dysregulation of macrophage numbers and accelerated branching morphogenesis (Wilson et al., 2017). The chemokines scavenged by ACKR2 are ligands for the signalling chemokine receptors CCR1, CCR2, CCR3, CCR4 and CCR5 (Fig. 1) (Nibbs and Graham, 2013; Bachelerie et al., 2014). It is likely, therefore, that the effects of ACKR2 on mammary gland development are indirect, and a consequence of the regulation of levels of chemokines capable of modulating macrophage function via one of these five receptors. Curiously, the dominant monocyte recruitment receptor, CCR2, does not control the rate of branching morphogenesis in the mammary gland (Wilson et al., 2017; Jäppinen et al., 2019), and mammary gland macrophages do not express CCR4 (Wilson et al., 2017). Together, this suggests that the phenotype seen in *Ackr2*^−/−^ mammary glands is a consequence of altered responses through CCR1, CCR3 or CCR5. The purpose of this study was to determine which of these three receptors is the reciprocal partner of ACKR2, in the regulation of branching morphogenesis in the developing mammary gland.

Here, we identify CCR1, and its ligand CCL7, as key regulators working with ACKR2 in a reciprocal manner to regulate macrophage numbers, and branching morphogenesis, in the developing mammary gland. Collectively, this study sheds important light on the regulation of macrophage dynamics during virgin mammary gland development.

## RESULTS

### Ductal branching in the pubertal mammary gland is regulated by CCR1

**Fig. 2.**
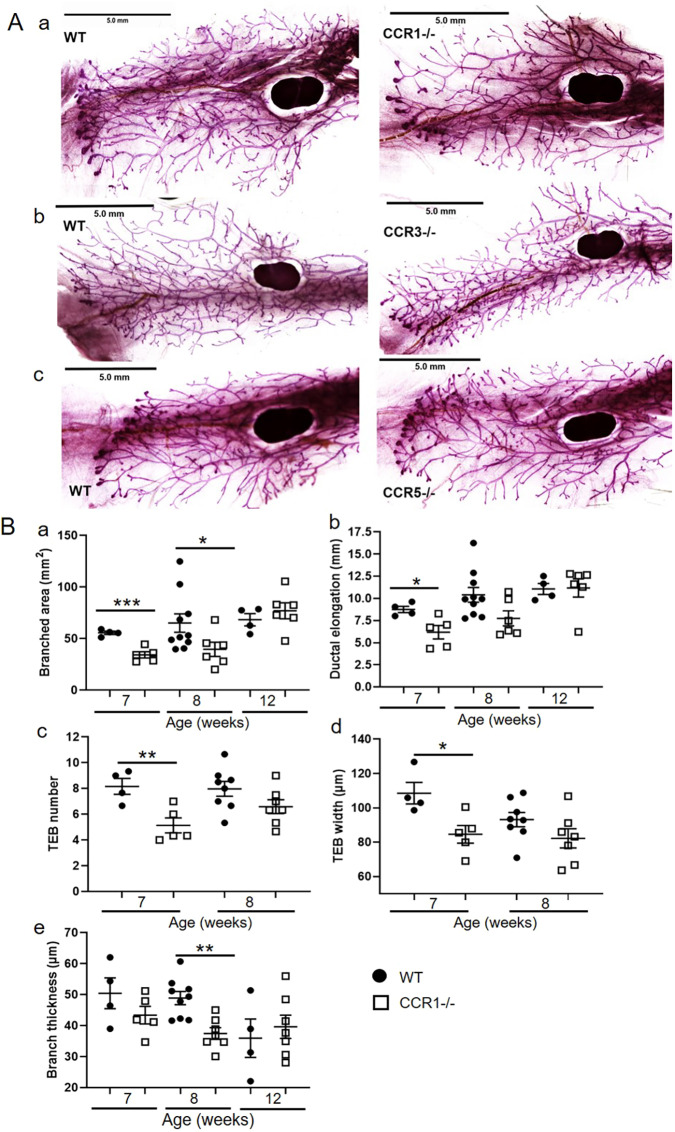
**Ductal branching in the pubertal mammary gland is regulated by CCR1.** (A) Representative carmine alum whole-mount images of late pubertal (7-week-old) virgin mammary glands from (a) wild-type and *Ccr1*^−/−^, (b) wild-type and *Ccr3*^−/−^, and (c) wild-type and *Ccr5*^−/−^ mice. (B) Branching morphogenesis was quantified in 7- (wild type, *n*=4; *Ccr1*^−/−^, *n*=5), 8- (wild type, *n*=10; *Ccr1*^−/−^, *n*=7) and 12- (wild type, *n*=4; *Ccr1*^−/−^, *n*=7) week-old mammary glands using ImageJ by measuring the following. (a) The area of branching from the inguinal lymph node: 7 weeks, two-tailed *t-*test (***P=0.0007); 8 weeks, Mann–Whitney test (*P=0.0312). (b) Ductal elongation, measured from the middle of the inguinal lymph node to the furthest edge of ductal outgrowth: 7 weeks, two-tailed *t-*test (*P=0.026). (c) The number of TEBs, determined as the average number from at least two individual fields of view (FOV) (5×) per gland: 7 weeks, two-tailed *t-*test (**P=0.0093). (d) The average width of all TEBs, determined from at least two FOV (5×) per gland: 7 weeks, two-tailed *t-*test (*P=0.0201). (e) Branch thickness, determined as the average of three measurements from six FOV (5×) per gland: 8 weeks, two-tailed *t-*test (**P=0.0015). Significantly different results are indicated. Scale bars: 5 mm. Data are mean±s.e.m.

To determine involvement of CCR1, CCR3 and CCR5 in the regulation of ductal branching morphogenesis in the mammary gland, we analysed carmine alum-stained wholemounts of mammary glands from 7-week-old wild-type, *Ccr1*^−/−^, *Ccr3*^−/−^ and *Ccr5*^−/−^ mice (Fig. 2Aa-c). The individual receptor-deficient mice have different genetic backgrounds; therefore, mice from each strain were compared to their specific wild type (Dyer et al., 2019). Quantitative analysis of the wholemounts indicated that branched area, ductal elongation, TEB number and width were unaffected in *Ccr3*^−/−^ and *Ccr5*^−/−^ mice (Fig. 2Ab,c, Fig. S1). In contrast, *Ccr1*^−/−^ mice exhibited delayed mammary gland development with decreased branched area at 7 and 8 weeks, reduced ductal elongation and decreased number and width of TEBs at 7 weeks (Fig. 2Aa and Fig. 2Ba-d). In addition, in comparison with wild-type mice, *Ccr1*^−/−^ mice had thinner branches at 8 weeks (Fig. 2Be). This was not seen for *Ccr3*^−/−^ or *Ccr5*^−/−^ mice (Fig. S1E). As observed for *Ackr2*^−/−^ mice, by 12 weeks, when TEBs have regressed and ductal outgrowth is completed, branched area and ductal elongation are equivalent between wild-type and *Ccr1*^−/−^ mice (Fig. 2Ba,b). Importantly, the onset of puberty, as assessed by vaginal opening, was normal in *Ccr1*^−/−^ mice (Table S1). Together, these data show that CCR1 regulates mammary gland development at a time point coincident with ACKR2 function in the same context.

Of note, in contrast to *Ackr2*^−/−^ mice, no difference was observed in the distance between, or density of, branches in wild-type and *Ccr1*^−/−^ mammary glands at any of the time points investigated (Fig. S2). This suggests that CCR1 does not regulate the density, but the spread of the ductal network.

Importantly, previous publications have suggested potential redundancy in roles for CCR1, CCR3 and CCR5 *in vivo* (Mantovani, 1999; Schall and Proudfoot, 2011). Although we have shown this not to be the case in acute inflammation (Dyer et al., 2019), we have not examined potential receptor redundancy in the context of mammary gland development. Therefore, to test for any potential redundancy between the CCRs, mammary gland wholemounts were obtained for iCCR^−/−^ mice, which have a compound deletion of CCR1, CCR2, CCR3 and CCR5 (Dyer et al., 2019). As observed in the absence of CCR1, iCCR^−/−^ mice display similar delayed development at 7 weeks, as demonstrated by reduced TEB number (Fig. S1C). No additional combinatorial effects of the receptors were observed, indicating that CCR1 is a non-redundant regulator of mammary gland development.

### CCR1 and ACKR2 are expressed surrounding epithelium in the mammary gland

**Fig. 3.**
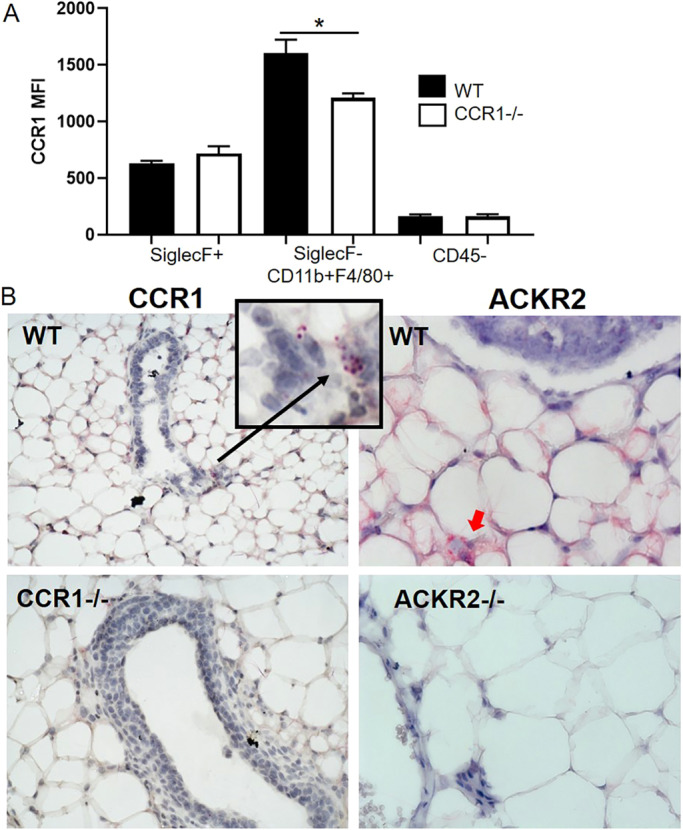
**CCR1 and ACKR2 are expressed surrounding epithelium in the mammary gland.** (A) Flow cytometry analysis of CCR1 expression by enzymatically digested wild-type (black bars, *n*=6) and *Ccr1*^−/−^ (white bars, *n*=4) mammary gland cells: CD45+ SiglecF+, CD45+SiglecF-CD11b+F480+ and CD45−. (B) RNAscope *in situ* hybridisation of CCR1 (highlighted by a black arrow) and ACKR2 (highlighted by a red arrow) in the developing virgin mammary gland of WT, *Ccr1*^−/−^ and *Ackr2*^−/−^ mice. Significantly different results are indicated: two-tailed *t-*test, *P=0.0305. Data are mean±s.e.m.

We next examined the expression patterns of CCR1 and ACKR2 within the developing mammary gland during late puberty. We used flow cytometry to identify the cell type(s) expressing CCR1 within the mammary gland. As currently available antibodies to murine CCR1 are of limited quality, we included cells from *Ccr1*^−/−^ mice as a control. This analysis demonstrated that CCR1 is detectable only on macrophages (CD45+SiglecF-CD11b+F4/80+) within the mammary gland (Fig. 3A) and further *in situ* hybridisation showed the CCR1+ cells to be intimately associated with the ductal epithelium (Fig. 3B). In contrast to macrophages, eosinophils (CD45+ SiglecF+) and stromal and epithelial (CD45−) cells did not express CCR1 (Fig. 3A). We next examined ACKR2 expression in the mammary gland. Previously, we have shown that ACKR2 is expressed by stromal fibroblasts in the developing virgin mammary gland (Wilson et al., 2017). Here, we have used *in situ* hybridisation to locate expression of ACKR2 to stromal cells in the vicinity of the ductal epithelium. Importantly no *in situ* hybridisation signals were seen in the stroma of *Ccr1*^−/−^ or *Ackr2*^−/−^ mammary glands (Fig. 3B). These data therefore demonstrate that CCR1 and ACKR2 are expressed by distinct cell types surrounding TEBs in the developing mammary gland.

### Oestrogen induces CCR1 expression on macrophages

**Fig. 4.**
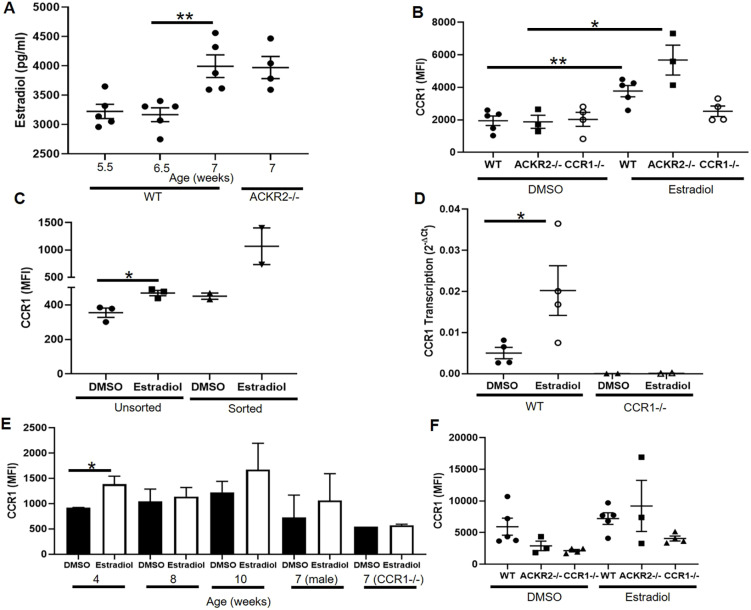
**Oestrogen induces CCR1 expression on macrophages.** (A) Oestradiol levels in plasma from 5.5-week-old wild type (*n*=5), 6.5-week-old wild type (*n*=5), 7-week-old wild type (*n*=5) and 6.5-week-old *Ackr2*^−/−^ (*n*=4). Two-tailed *t-*test (**P=0.0064). (B) CCR1 expression in CD11b+F4/80+ cells in response to DMSO and 50 µg/ml oestradiol in wild type (*n*=5), *Ackr2*−/− (*n*=3) and *Ccr1*^−/−^ (*n*=4). Wild type, two-tailed *t-*test (**P=0.004); *Ackr2*^−/−^, two-tailed *t-*test (*P=0.0194). (C) Unsorted (*n*=3) and CD11b+F4/80+ FACS-sorted cells (*n*=2) from the mammary gland. Two-tailed *t-*test (*P=0.0217). (D) Transcription of CCR1 by CD11b+F4/80+ FACS-sorted cells from wild-type (*n*=4) and *Ccr1*^−/−^ (*n*=2) mice, in response to DMSO or oestradiol. Two-tailed *t-*test (*P=0.0494). (E) CCR1 expression in CD11b+F4/80+ cells from female wild-type mammary glands at 4, 8 and 10 weeks of age, and in female 7-week-old *Ccr1*^−/−^ mice and male wild-type inguinal fat pads from 7-week-old mice, in response to DMSO and 50 µg/ml 17β-oestradiol (each group, *n*=3). Two-tailed *t-*test (*P=0.04). (F) CCR1 expression in CD11b+F4/80+ cells from the peritoneum: wild type (*n*=5), *Ackr2*^−/−^ (*n*=3) and *Ccr1*^−/−^ (*n*=4). Significantly different results are indicated. Data are mean±s.e.m.

We next examined regulation of CCR1 expression on mammary gland macrophages. Oestrogen is essential for mammary gland development and ductal epithelial growth and proliferation (Hovey and Trott, 2002). ELISA-based analysis of oestradiol levels in the plasma of the developing mouse indicated that its production rises over the same time frame in which we observe altered ductal development in *Ackr2*^−/−^ and *Ccr1*^−/−^ mammary glands (Fig. 4A). Notably, there was no difference in the levels of oestradiol between wild-type and *Ackr2*^−/−^ mice, suggesting that the accelerated branching in *Ackr2*^−/−^ mice is not caused by increased levels of oestrogen. To determine whether oestrogen regulates CCR1 expression on mammary gland macrophages, we enzymatically digested mammary glands and exposed the cells to DMSO (vehicle control) or 17β-oestradiol for 1h at 37°C. CCR1 expression was analysed by flow cytometry and shown to increase on CD45+ CD11b+F4/80+ macrophages in response to 17β-oestradiol (Fig. 4B). There was no significant difference between the level of CCR1 expression on wild-type and *Ackr2*^−/−^ macrophages after exposure, indicating that ACKR2 does not regulate this process.

To determine whether this was a direct effect of oestradiol on mammary gland macrophages, CD11b+F4/80+ cells were isolated by FACS. In the absence of other cell types, CCR1 expression was increased following exposure to 17β-oestradiol, indicating that oestrogen-mediated induction of CCR1 results from a direct effect on mammary gland macrophages (Fig. 4C). In addition, we showed that transcription of CCR1 mRNA by purified CD11b+F4/80+ cells is increased in response to oestradiol, suggesting that CCR1 is being synthesised *de novo* (Fig. 4D).

Notably, upregulation of CCR1 on macrophages in response to oestradiol is age dependent, as there is no difference in CCR1 expression in mice 8 weeks or older (Fig. 4E). In addition, 17β-oestradiol has no effect on macrophages isolated from the male fat pad or the peritoneum of pubertal female mice (Fig. 4E,F). Taken together, this suggests that the effect of oestrogen on CCR1 expression is restricted to pubertal mammary gland macrophages and limited to the key developmental time frame we have identified.

### Chemokine levels are altered in the absence of CCR1 and ACKR2

**Fig. 5.**
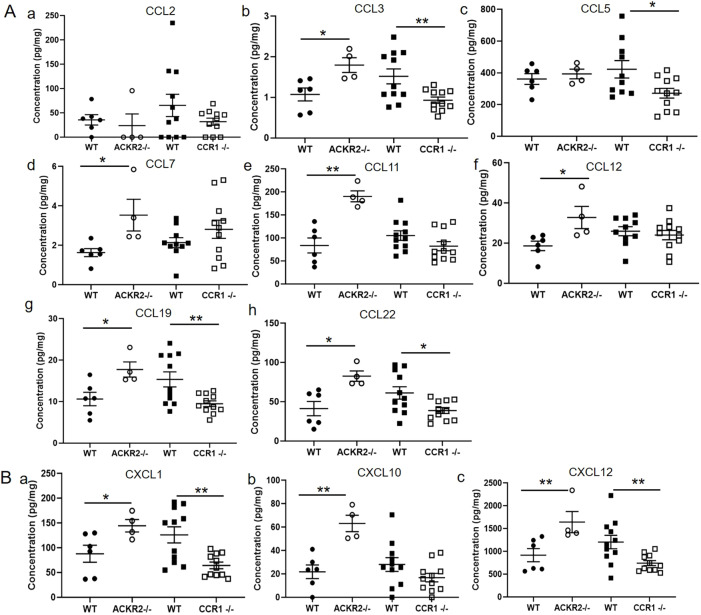
**Chemokine levels are altered in the absence of ACKR2 and CCR1.** Multiplex measurement of protein concentration of (A) inflammatory CC chemokines in whole mammary gland homogenates: (a) CCL2; (b) CCL3, two-tailed *t-*test, *Ackr2*^−/−^ (*P=0.0183) and *Ccr1*^−/−^ (**P=0.0082); (c) CCL5, two-tailed *t-*test, *Ccr1*^−/−^ (*P=0.0235); (d) CCL7, two-tailed *t-*test, *Ackr2*^−/−^ (*P=0.024); (e) CCL11, two-tailed *t-*test, *Ackr2*^−/−^ (**P=0.0014); (f) CCL12, two-tailed *t-*test, *Ackr2*^−/−^ (*P=0.0279); (g) CCL19, two-tailed *t-*test, *Ackr2*^−/−^ (*P=0.0216) and *Ccr1*^−/−^ (**P=0.0065); and (h) CCL22, two-tailed *t-*test, *Ackr2*^−/−^ (*P=0.0108) and *Ccr1*^−/−^ (*P=0.0184). (B) CXC chemokines: (a) CXCL1, two-tailed *t-*test, *Ackr2*^−/−^ (*P=0.0437) and *Ccr1*^−/−^ (**P=0.0023); (b) CXCL10, two-tailed *t-*test, *Ackr2*^−/−^ (**P=0.0020); (c) CXCL12, Mann–Whitney test, *Ackr2*^−/−^ (**P=0.0095), two-tailed *t-*test, *Ccr1*^−/−^ (**P=0.0086); wild type (CCR1) *n*=11, *Ccr1*^−/−^ *n*=11, wild type (ACKR2) *n*=6 and *Ackr2*^−/−^ *n*=4. Significantly different results are indicated. Data are mean±s.e.m.

To identify the specific chemokines involved in regulating mammary gland development through CCR1 and ACKR2, multiplex protein analysis of mammary gland lysates was carried out. In keeping with our previous data, we showed that, in the absence of scavenging by ACKR2, the chemokines CCL7, CCL11 and CCL12 accumulate in the mammary gland at 7 weeks (Fig. 5A) (Wilson et al., 2017). The current analysis further revealed elevated levels of the ACKR2 ligands CCL3 and CCL22 in the *Ackr2*^−/−^ mammary gland at 7 weeks (Fig. 5A). Notably, other key ACKR2 ligands associated with monocyte and macrophage migration, i.e. CCL2 and CCL5, are unchanged in the *Ackr2*^−/−^ mammary gland (Fig. 5A). Importantly, there were no significant differences in the levels of these chemokines in lysates obtained from male wild-type and *Ackr2*^−/−^ inguinal fat pads, indicating that the changes observed in female lysates are specifically associated with the mammary gland (Fig. S3). In *Ccr1*^−/−^ mice, the levels of CCL7, CCL11 and CCL12 were unchanged, suggesting that ACKR2 is functional in these mice and able to scavenge chemokines normally. A number of chemokines, including CCL19, CXCL1 and CXCL12, which are not ligands for either ACKR2 or CCR1, are increased in *Ackr2*^−/−^ mice and decreased in *Ccr1*^−/−^ mice (Fig. 5). It is likely that their altered levels reflect variation in the numbers of chemokine-expressing immune cells or the extent of epithelial cell branching within the mammary gland. Bioinformatic analysis of mammary epithelial cell single cell data reveals that CXCL1, CXCL10 and CXCL12 are produced by epithelial cells (Fig. S4; Bach et al., 2017).

### CCR1 and ACKR2 reciprocally regulate CD206+ macrophages within the mammary gland

**Fig. 6.**
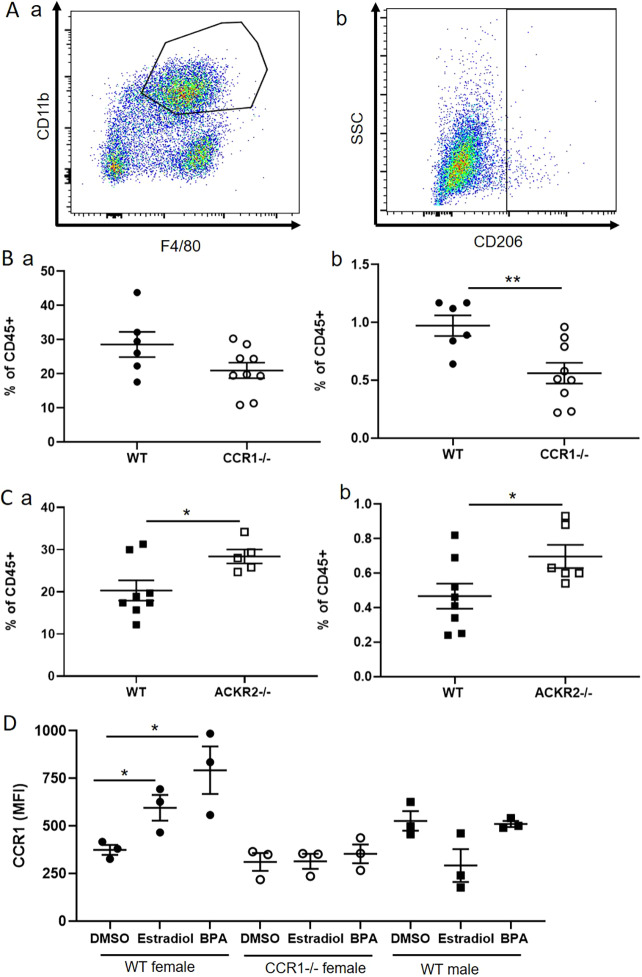
**CCR1 and ACKR2 reciprocally regulate CD206+ macrophages within the mammary gland.** (A) Flow cytometry was used to reveal (a) CD11b+F4/80+ and (b) SiglecF- F4/80+ CD206+ macrophages, within the CD45+ compartment of the 6.5-week-old developing mammary gland. (B) Flow cytometry of (B) wild-type (*n*=6) and *Ccr1*^−/−^ (*n*=9) mammary glands to reveal and determine the percentage of (a) CD11b+F4/80+ cells and (b) SiglecF- F4/80+ CD206+ cells; two-tailed *t-*test (**P=0.0079). (C) Flow cytometry of wild-type (*n*=8) and *Ackr2*^−/−^ (*n*=5) mammary gland cells was carried out for (a) CD11b+F4/80+ cells, two-tailed *t-*test (*P=0.0339) and (b) SiglecF- F4/80+ CD206+ cells, two-tailed *t-*test (*P=0.0439). (D) CCR1 expression by SiglecF- F4/80+ CD206+ cells in response to DMSO and 50 µg/ml 17β-oestradiol and bisphenol A (BPA); female wild type and *Ccr1*^−/−^, and male wild type (each group, *n*=3). Two-tailed *t-*test, oestradiol (*P=0.0381) and BPA (*P=0.0307). Significantly different results are indicated. Data are mean±s.e.m.

Reciprocal regulation of leukocyte dynamics by CCR1 and ACKR2 in the developing mammary gland should be reflected in complementary changes in levels of key cellular populations in *Ccr1*^−/−^ and *Ackr2*^−/−^ mice. We detected no significant differences in the lymphocyte populations or in non-macrophage myeloid cell populations investigated. However, differences in a key macrophage population were identified. To investigate the effects of CCR1 deficiency on macrophage levels in the mammary gland, flow cytometry of enzymatically digested 6.5-week-old wild-type and *Ccr1*^−/−^ glands was carried out. The gating strategy employed is described in Fig. S5. *Ccr1*^−/−^ mice displayed no significant differences in the bulk macrophage population (CD45+CD11b+F4/80+) (Fig. 6Aa,Ba). However, we detected a significant decrease in the percentage of a small population of macrophages expressing CD206 (mannose receptor) (CD45+SiglecF-F4/80+CD206+) in *Ccr1*^−/−^ mice (Fig. 6Ab,Bb). Analysis of *Ackr2*^−/−^ mice revealed a complementary phenotype to *Ccr1*^−/−^ mice in that they displayed an increase in the percentage of macrophages in the mammary gland population and specifically of the CD206+ macrophage subset (Fig. 6C). To determine whether CD206+ macrophages are recruited later in *Ccr1*^−/−^ mice, we carried out flow cytometric analysis at the later time points of 7, 8 and 12 weeks (Fig. S6). The number of CD206+ macrophages was not increased in *Ccr1*^−/−^ mice at any of the time points investigated.

Finally, we examined the effects of oestrogen on the CD206+ macrophage population. Our data show that CCR1 expression was also increased on the surface of CD206+ macrophages in response to both 17β-oestradiol and the oestrogen mimic bisphenol A (BPA) (Fig. 6D). No effect of oestrogen on CCR1 expression was observed in male macrophages (Fig. 6D).

Thus, a key population of CD206+ macrophages are reciprocally regulated by ACKR2 and CCR1. Importantly, CD206+ mammary gland macrophages have previously been implicated in branching morphogenesis (Jäppinen et al., 2019) and we propose that ACKR2 and CCR1 reciprocally control this population to coordinate branching morphogenesis in the pubertal mammary gland.

### CCL7 regulates CD206+ macrophages and branching morphogenesis

**Fig. 7.**
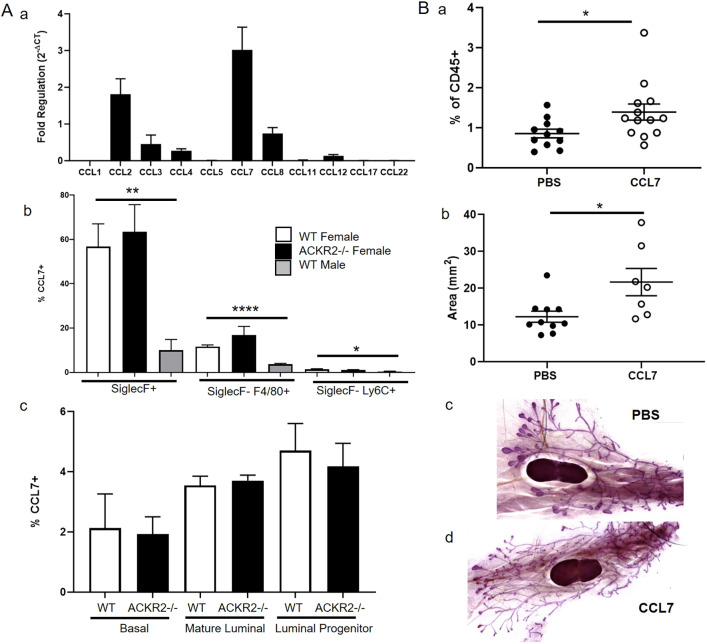
**CCL7 controls CD206+ macrophages and branching morphogenesis.** (A) (a) Transcription of inflammatory chemokines by purified F4/80+ cells (*n*=3). CCL7 is produced in the mammary gland by (b) immune cells, including SiglecF+, SiglecF-F4/80+ and SiglecF-Ly6C+ female wild-type (*n*=6), female *Ackr2*^−/−^ (*n*=4) and male wild-type (*n*=7) cells; two-tailed *t-*test, SiglecF+ (**P=0.0012) and SiglecF-F4/80+ (****P<0.0001); and Mann–Whitney, SiglecF-Ly6C+ (*P=0.0198). (c) CCL7 production by epithelial cells, mature luminal (EpCAM+ CD49f−), progenitor luminal (EpCAM+ CD49f+) and basal (EpCAM− CD49f+) cells: female wild type (*n*=6) and *Ackr2*^−/−^ (*n*=4). (B) 3 days after subcutaneous administration of PBS or 2 µg CCL7 at 6 weeks, (a) the percentage of SiglecF-F4/80+CD206+ cells was measured by flow cytometry (PBS, *n*=11; CCL7, *n*=13), Mann–Whitney test (*P=0.0289); and (b) the area of branching was measured using ImageJ (PBS, *n*=10; CCL7, *n*=7), Mann–Whitney test (*P=0.0185). (c,d) Representative images of wholemounts from PBS- and CCL7-injected mice. Significantly different results are indicated. Data are mean±s.e.m.

Of the chemokines detected within the mammary gland, CCL7 is of particular interest as it is shared between CCR1 and ACKR2 (Fig. 1), and is elevated in the pubertal mammary glands of *Ackr2*^−/−^ mice (Fig. 5Ad) (Wilson et al., 2017). In addition, qRT-PCR analysis also revealed that CCL7 is transcribed, by purified F4/80+ cells, at higher levels than other ACKR2 ligands (Fig. 7Aa). We therefore investigated its expression and function in the mammary gland. Using flow cytometry, intracellular staining revealed that CCL7 is produced by immune cells, including SiglecF+ eosinophils, SiglecF− F4/80+ macrophages and SiglecF-Ly6C+ monocytes (Fig. 7Ab). For each cell type, a markedly higher percentage of cells obtained from the female mammary gland produced CCL7, than from male fat pad cells. Notably, around 60% of female SiglecF+ cells produced CCL7 compared with 10% of male cells (Fig. 7Ab). The percentage of CCL7+ cells was unaffected in the absence of ACKR2 (Fig. 7Ab). CCL7 is also produced by CD45− epithelial cells: mature (EpCAM+ CD49f−) and progenitor luminal (EpCAM+ CD49f+), and basal (EpCAM− CD49f+) cells (Fig. 7A c, Fig. S5B). Furthermore, bioinformatic analysis confirmed that CCL7 is produced by epithelial cells, including basal, luminal and myoepithelial cells (Fig. S4) (Bach et al., 2017).

Given the notable CCL7 expression in the mammary gland, we next directly tested its potential role in mammary gland development. PBS or 2 µg of CCL7 was administered subcutaneously at the site of the mammary fat pad at the key time point of 6 weeks. After 3 days, mammary glands were harvested for cellular analysis by flow cytometry and carmine alum whole-mount analysis. CCL7 administration alone was sufficient to increase the percentage of CD206+ macrophages, and the area of branching within the mammary gland (Fig. 7B). These data confirm that elevated levels of CCL7, as observed in *Ackr2*^−/−^ mice, lead to increased numbers of CD206+ macrophages in the mammary gland and accelerated branching. To determine the specificity of this interaction, we investigated the effect of other chemokines on branching and macrophage recruitment. CCL3 or CCL11 administration did not increase branching or the number of CD206+ macrophages in the mammary gland (Fig. S7). Thus, the CCL7/CCR1/ACKR2 signalling axis appears to be specific.

Overall, these data demonstrate a role for CCL7, a ligand shared by CCR1 and ACKR2, in branching morphogenesis. Lending further support to this conclusion is the fact that bioinformatic interrogation of the precocious puberty (CTD Gene-Disease Associations) dataset, using Harmonizome (Rouillard et al., 2016), revealed that CCL7 and ACKR2 are both associated with precocious puberty in children, with standardised values of 1.25588 (*P*=0.09) and 1.02634 (*P*=0.011), respectively.

## DISCUSSION

**Fig. 8.**
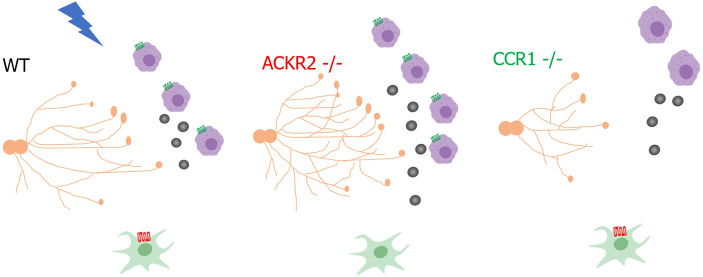
**Proposed mechanism by which chemokine receptors CCR1 and ACKR2 coordinate mammary gland development.** Oestrogen (blue lightning) increases CCR1 expression on macrophages (purple) during puberty, and stromal fibroblast (green)-expressed ACKR2 modulates levels of CCL7 (grey circles) to control the movement of CCR1+ macrophages to the ductal epithelium (orange). Schematic image was created with BioRender.

The importance of macrophages in controlling developmental processes is well known (Wynn et al., 2013). The role of chemokines and their receptors, which provide molecular cues to guide and position macrophages during development, is an emerging area of research (Lee et al., 2014; Wilson et al., 2017). Previously, we revealed that the scavenging atypical chemokine receptor ACKR2 controlled macrophages in the mammary gland through a CCR2-independent pathway (Wilson et al., 2017). Here, we have revealed a previously unknown immunological mechanism, whereby ACKR2 and the inflammatory chemokine receptor CCR1 interact with their shared ligand CCL7 to coordinate the levels of CD206+ macrophages and, thus, the extent of branching morphogenesis in the pubertal mammary gland. Importantly, administration of CCL7 alone was able to increase the percentage of CD206+ macrophages within the mammary gland and drive accelerated branching morphogenesis. We propose that, in *Ccr1*^−/−^ mice, although CCL7 levels are unaltered, macrophages are unable to sense and respond to the ligand without the cognate receptor, leading to delayed branching (Fig. 8).

Previously, it was thought that all mammary gland macrophages, at rest and pathologically, were derived from the bone marrow (Coussens and Pollard, 2011). In our previous study, we showed that branching was unaltered in the absence of CCR2, indicating that the macrophage population responsible for promoting branching morphogenesis was unlikely to be bone marrow derived (Wilson et al., 2017). Recently, a novel CD206+ macrophage population has been identified in the mammary gland, which is unaffected in the absence of CCR2, but reduced in *Plvap*^−/−^ mice, which have reduced numbers of foetal-derived macrophages (Jäppinen et al., 2019). Branching is severely impaired in these mice, suggesting that foetal-derived macrophages play a key role in promoting branching morphogenesis (Jäppinen et al., 2019). We believe that the macrophage population identified in our study may be derived from the same embryonic population (Jäppinen et al., 2019). Importantly, the effect of CCR1 deficiency on mammary gland development is less pronounced than was observed for complete loss of macrophages (Gouon-Evans et al., 2000). This suggests that macrophages that do not express CCR1, CCR2, CCR3 or CCR5, such as those recruited through CX3CR1 may also be important in regulating branching. A recent study has identified a population of ductal macrophages that express CX3CR1 and have important roles in surveillance and tissue remodelling (Dawson et al., 2020).

Eosinophils are known to be important in controlling mammary gland development as branching complexity is reduced in CCL11-deficient mice, which have decreased numbers of eosinophils (Gouon-Evans et al., 2000). Here, we have shown that eosinophils are an important source of CCL7 for macrophages. *Ccr3*^−/−^ mice also have reduced numbers of eosinophils (Dyer et al., 2019). Here, we reveal that the extent of branching is unaffected in *Ccr3*^−/−^ mice, suggesting that eosinophils do not directly control the extent of branching in the mammary gland.

CCR1 is an inflammatory chemokine receptor that is expressed by immune cells, and has been shown to be important in a number of pathologies, including sepsis, viral infections, cancer and autoimmune disease (Domachowske et al., 2000; Katschke et al., 2001; Ness et al., 2004; Kitamura et al., 2015). To our knowledge, this is the first description of a key role for CCR1 in development. Of note, in the placenta, CCR1 has been shown to be expressed by human trophoblasts as they switch to an invasive phenotype (Sato et al., 2003). ACKR2 is highly expressed by placental trophoblasts, preventing excess levels of inflammatory chemokines from entering the foetus, from the mother's circulation, by a process of chemokine compartmentalisation (Teoh et al., 2014; Lee et al., 2019). As CCR1 expression has also been described in placental development, there could be wider implications of the interaction described in this study.

In the mouse, sexual maturity occurs at around 6 weeks (Topper and Freeman, 1980). Here, we report a marked increase in plasma oestradiol levels between 6.5 and 7 weeks. This is the key time point in ACKR2/CCR1-dependent regulation of branching morphogenesis. ACKR2 expression in the mammary gland specifically peaks at 6.5 weeks and branching begins to accelerate at this time point (Wilson et al., 2017). We show that 17β-oestradiol increases CCR1 expression on macrophages. However, this is restricted to pubertal mammary gland macrophages, as older female, male and peritoneal macrophages do not respond. In addition to 17β-oestradiol, the oestrogen mimic bisphenol A also increased CCR1 expression on CD206+ macrophages. This may be of concern as BPAs are widely found in the environment and could potentially alter the immune response, and the extent of branching in the mammary gland, in children during puberty. Previously, CCR1 expression on T cells was shown to be regulated by 17β-oestradiol (Mo et al., 2005). However, this is the first description of oestrogen-controlled CCR1 expression on macrophages. This observation could have implications for our understanding of diseases where females exhibit increased susceptibility. One example is rheumatoid arthritis, where CCR1 is also associated with pathology (Katschke et al., 2001; van Vollenhoven, 2009).

Understanding the molecular signals that guide the rate of branching morphogenesis in the mammary gland is highly important. Precocious puberty is a condition where puberty begins before the age of 8, with some girls developing breasts as early as 4. This results from early activation of the gonadotropic axis, leading to accelerated growth and bone maturation, but ultimately reduced stature (Carel et al., 2004). Potential risk factors include exposure to endocrine disrupters, obesity, stress and ethnicity (Cesario and Hughes, 2007; Lee et al., 2007; Meeker, 2012; Kelly et al., 2017). As mammary gland development is delayed in mice in the absence of CCR1, this could represent a novel therapeutic target to treat aspects of precocious puberty. Several CCR1 antagonists are available and have been used in a number of clinical trials (Lebre et al., 2011). In addition, early breast development leads to higher risks of breast cancer in later life (Bodicoat et al., 2014), and women with dense breasts are more likely to develop breast cancer (Nazari and Mukherjee, 2018). This can be related to poor detection by mammography as the branches mask the cancer, but may also be caused by genetic factors, parity and alterations in the breast stroma. Both ACKR2 and CCR1 have been shown to be important in the progression of breast cancer; therefore, understanding early interactions between these receptors could reveal key insights, which drive later pathology (Kitamura et al., 2015; Shin et al., 2017; Hansell et al., 2018).

In this study, we have uncovered a novel mechanism by which oestradiol upregulates CCR1 expression by pubertal mammary gland macrophages and stromal ACKR2 modulates levels of CCL7, to control the movement of the CCR1+ macrophages to the ductal epithelium. Overall, therefore, our data demonstrate that CCR1 and ACKR2 coordinately regulate mammary gland branching morphogenesis.

## MATERIALS AND METHODS

### Animals

Animal experiments were carried out under the auspices of a UK Home Office Project Licence and conformed to the animal care and welfare protocols approved by the University of Glasgow. C57BL/6 mice, *Ackr2*^−/−^ (Jamieson et al., 2005), *Ccr1*^−/−^, *Ccr3*^−/−^, *Ccr5*^−/−^ and iCCR^−/−^ (Dyer et al., 2019) mice were bred at the specific pathogen-free facility of the Beatson Institute for Cancer Research.

### Carmine alum wholemount

Carmine alum wholemounts were prepared as described previously (Wilson et al., 2017). Briefly, fourth inguinal mammary glands were spread onto Superfrost Plus slides (VWR) and fixed overnight in 10% neutral buffered formalin (NBF) (Leica) at 4°C. Glands were dehydrated for 1 h in distilled water, followed by 70% ethanol and 100% ethanol before overnight incubation in xylene (VWR international). Tissue was rehydrated by a 1 h incubation in 100% ethanol, 70% ethanol and distilled water, before staining in carmine alum solution overnight at room temperature [0.2% (w/v) carmine and 10 mM aluminium potassium sulphate (Sigma)]. Tissue was dehydrated again before overnight incubation in xylene. Finally, glands were mounted with DPX (Leica) and stitched bright-field images at 10× magnification were taken using an EVOS FL auto2 microscope (ThermoFisher). Ductal elongation, and branched area from the lymph node, were measured using ImageJ 1.52a (Schneider et al., 2012). Bright-field images at 5× magnification were obtained using the Zeiss Axioimager M2 with Zen 2012 software. The numbers of branches and branch thickness were counted as the average from three measurements from six individual fields of view (FOV) from each wholemount. TEBs were counted as the average from at least two FOV from each wholemount. Sample identities were hidden before measurements were taken.

### RNAscope *in situ* hybridisation

Mammary glands were fixed in 10% neutral buffered formalin at room temperature for 24-36 hours before being dehydrated using rising concentrations of ethanol and xylene, and paraffin embedded (Shandon citadel 1000, Thermo Shandon). Tissue was sectioned onto Superfrost plus slides (VWR) at 6 μm using a Microtome (Shandon Finesse 325 Microtome, Thermo Shandon). Slides were baked at 60°C for 1 h before pre-treatment. Slides were deparaffinised with xylene (twice for 5 min) and dehydrated with ethanol (twice for 1 min). Tissues were incubated with hydrogen peroxide for 10 min at room temperature, then boiled in antigen-retrieval buffer for 15 min. Slides were treated with protease plus for 30 min at 40°C. Slides were then hybridised using the RNAScope 2.5 Red Manual Assay (Advanced cell diagnostics) according to the manufacturer's instructions using the Mm-*Ccr1* and Mm-ACKR2 probes. Slides were mounted in DPX (Sigma Aldrich) and imaged on an EVOS FL Auto2microscope.

### Mammary gland digestion

The inguinal lymph node was removed from the fourth inguinal mammary gland, tissue was chopped, and enzymatic digestion was carried out in a 37°C shaking incubator at 200 rpm for 1 h, with 3 mg/ml collagenase type 1 (Sigma) and 1.5 mg/ml trypsin (Sigma) in 2 ml Leibovitz L-15 medium (Sigma). The suspension was shaken for 10 s before addition of 5 ml of L-15 medium supplemented with 10% foetal calf serum (Invitrogen) and centrifugation at 400 ***g*** for 5 min. Red blood cells were lysed using Red Blood Cell Lysing Buffer Hybri-Max (Sigma) for 1 min and washed in PBS. Cells were washed in PBS with 5 mM EDTA, resuspended in 2 ml 0.25% Trypsin-EDTA (Sigma) and incubated at 37°C for 2 min before addition of 5 ml of serum-free L-15 containing 1 μg/ml DNase1 (Sigma) for 5 min at 37°C. L-15 containing 10% FCS was added to stop the reaction and cells were filtered through a 40 μm cell strainer before a final wash in FACS buffer (PBS containing 1% FCS and 5 mM EDTA).

### Flow cytometry

Antibodies were obtained from BioLegend and used at a dilution of 1:200: CD45 (30-F11), CD11b (M1/70), F4/80 (BM8), SiglecF (S17007L), Ly6C (HK1.4), EpCAM (G8.8), CD49f(GoH3), CCR1 (S10450E) and CD206 (C068C2) were incubated for 30 min at 4°C. Dead cells were excluded using Fixable Viability Dye eFluor 506 (Thermo Fisher). Intracellular staining for CCL7 was carried out using 1 in 100 biotinylated CCL7 antibody (R&D Systems), Strepdavidin BV605 (BioLegend), and eBioscience intracellular fixation and permeabilisation buffer. Flow cytometry was performed using an LSRII or Fortessa (BDBiosciences) and analysed using FlowJo V10. FACS sorting was carried out using a BD FACS ARIA III.

### Proteomic analysis

The inguinal lymph node was removed from the fourth inguinal mammary gland, tissue was chopped, frozen in liquid nitrogen, crushed with a mortar and pestle, and resuspended in distilled H_2_O containing protease inhibitors (Pierce). Protein levels were determined using a custom-designed Magnetic Luminex Multiplex assay (R&D Systems), as described in the manufacturer's instructions, and read with a Bio-Rad Luminex-100 machine. Data were normalised to the protein concentration of tissue samples, determined by a BCA assay (Pierce).

### Subcutaneous administration of chemokines

CCL7, CCL3 or CCL11 (2 µg in 200 µl PBS) (R&D Systems) was injected subcutaneously into mice at 6 weeks of age. After 3 days, mice were culled, and mammary glands were excised and processed for whole-mount and cellular analysis.

### 17β-Oestradiol assays

Fourth inguinal mammary glands were digested to obtain single cell suspensions. Cells were plated at 0.5-1×10^5^ cells in a 96 well plate in L-15 media containing 10% FCS and exposed to DMSO (vehicle control) or 50 µg/ml 17-β-oestradiol or bisphenol A (Sigma) for 1 h at 37°C, 5% CO_2_. Where CD11b+ F4/80+ cells were FACS sorted, 1×10^4^ sorted cells per well were exposed to DMSO or oestradiol. The level of 17-β-oestradiol in plasma samples was determined using the Estradiol parameter kit (R&D Systems), as described in the manufacturer's instructions.

### Transcriptional analysis

Cells were lysed using Buffer RLT and processed using a microRNeasy kit (Qiagen) as described previously (Wilson et al., 2017). Transcription levels were determined by quantitative real-time polymerase chain reaction (qRT-PCR) using primers to detect CCR1 and GAPDH (Dyer et al., 2019). Fold regulation was determined using the 2^(−ΔCT)^ method, where ΔC_T_ is calculated as C_T target_−C_T normaliser_. Normalisation was carried out using GAPDH. ACKR2 ligands were detected using the mouse Chemokine and Chemokine Receptor RT^2^ profiler PCR array (Qiagen) as described previously (Wilson et al., 2017).

### Bioinformatic analysis

Chemokine expression by epithelial cells was determined by searching the data repository from Bach et al. (2017) at: https://marionilab.cruk.cam.ac.uk/mammaryGland/.

### Statistical analysis

Data were analysed using GraphPad Prism 8.1.2. Normality was assessed using Shapiro Wilk and Kolmogorov–Smirnov tests. For data with normal distribution, two-tailed unpaired *t*-tests were used. Where data were not normally distributed, Mann–Whitney tests were used. Significance was defined as *P*<0.05. Error bars indicate standard error of the mean (s.e.m.).

## Supplementary Material

Supplementary information

Reviewer comments
