# Supplementary material for: Chemokine receptors coordinately regulate macrophage dynamics and mammary gland development
Source: Development. 2020 Jun 17;147(12):dev187815. doi: 10.1242/dev.187815 (PMC7328164; doi:10.1242/dev.187815)
Supplement: Supplementary information [file develop-147-187815-s1.pdf]

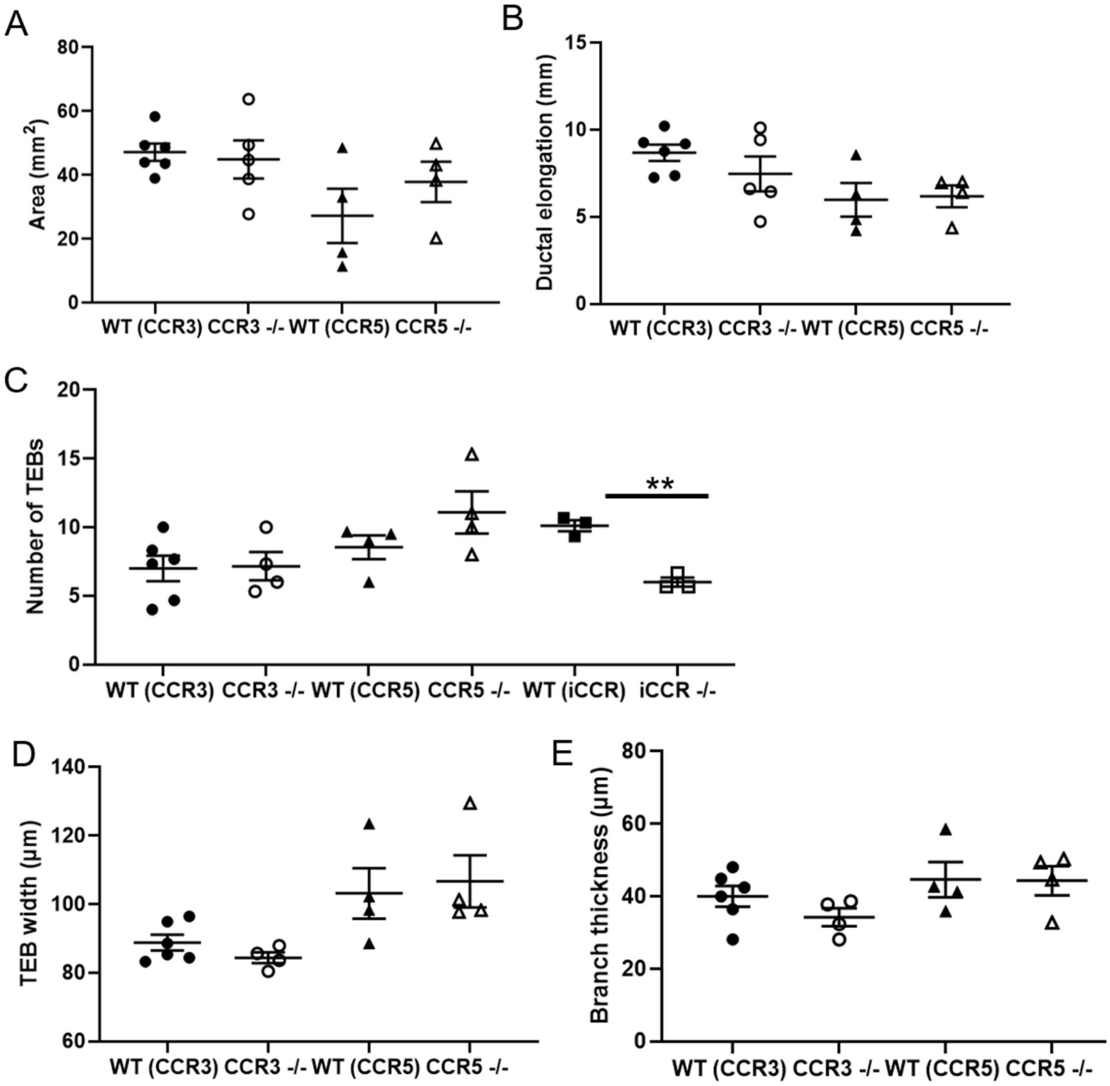

Figure S1: CCR3 and CCR5 do not control the extent of branching morphogenesis. Branching morphogenesis in carmine alum whole mounts were quantified in 7 weeks old mammary glands; (WT (CCR3) n=6, CCR3-/- n= 5; WT (CCR5) n=4, CCR5-/- n= 4). **(A)** the area of branching from the inguinal lymph node, **(B)** ductal elongation, measured from the middle of the inguinal lymph node to the furthest edge of ductal outgrowth. **(C)** The number of TEBs, was determined as the average number from at least 2 individual fields of view (FOV) (5×) per gland. WT (iCCR) n=3, iCCR-/- n= 3), two-tailed t-test (p=0.0014). **(D)** The average width of all TEBs was determined from at least 2 F.O.V (5x) per gland. **(E)** Branch thickness was determined as the average of 3 measurements from 6 x F.O.V (5x) per gland. Error bars represent S.E.M.

Development • Supplementary information

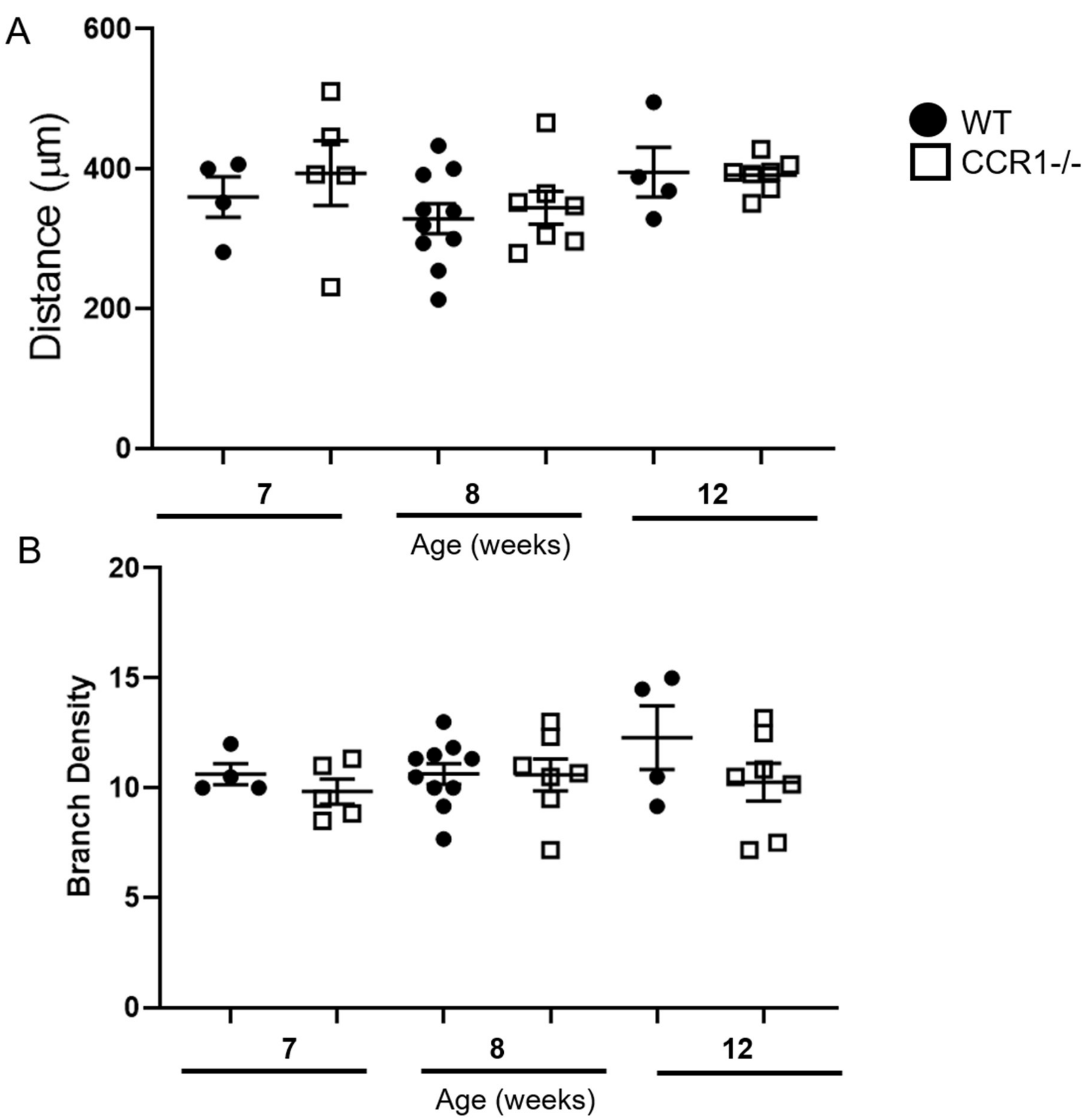

Figure S2: Branch density is unaffected in CCR1-/- mice. Branching morphogenesis in carmine alum whole mounts were quantified in 7 (WT n=4, CCR1-/- n= 5), 8 (WT n=10, CCR1-/- n= 7) and 12 (WT n=4, CCR1-/- n= 7) week mammary glands; in terms of **(A)** the distance between branches, and **(B)** the number of branches in a 5 x F.O.V. Each data point represents the average of 3 measurements from 6 individual F.O.V. per gland. Error bars represent S.E.M.

Development • Supplementary information

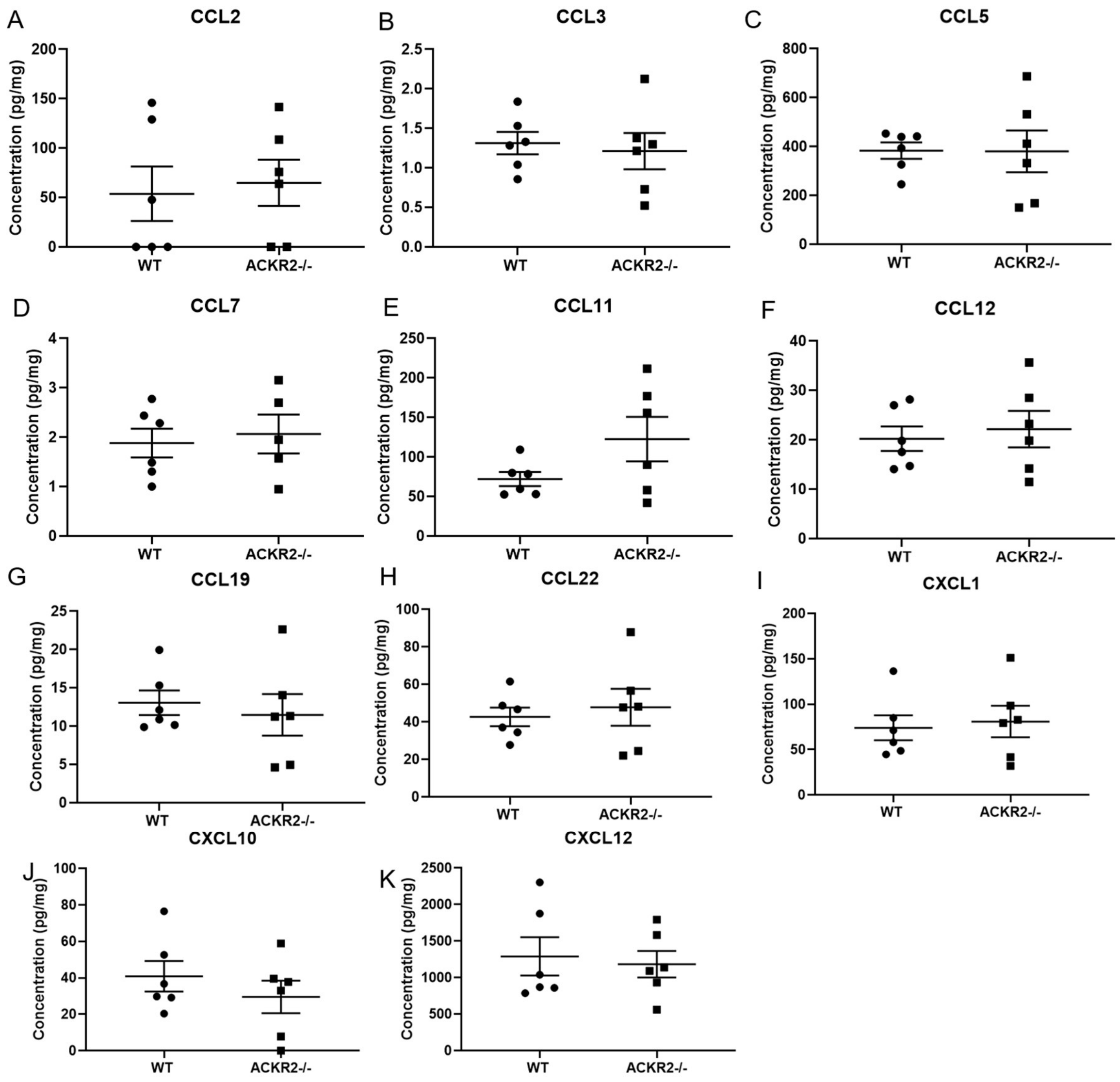

Figure S3: Chemokine levels in the male fat pad are unaffected in the absence of ACKR2. Multiplex measurement of protein concentration of **(A)** CCL2, **(B)** CCL3, **(C)** CCL5, **(D)** CCL7, **(E)** CCL11, **(F)** CCL12, **(G)** CCL19, **(H)** CCL22, **(I)** CXCL1, **(J)** CXCL10 and **(K)** CXCL12 in whole fat fad homogenates. WT n=6, and ACKR2-/- n=6. Error bars represent S.E.M.

Development • Supplementary information

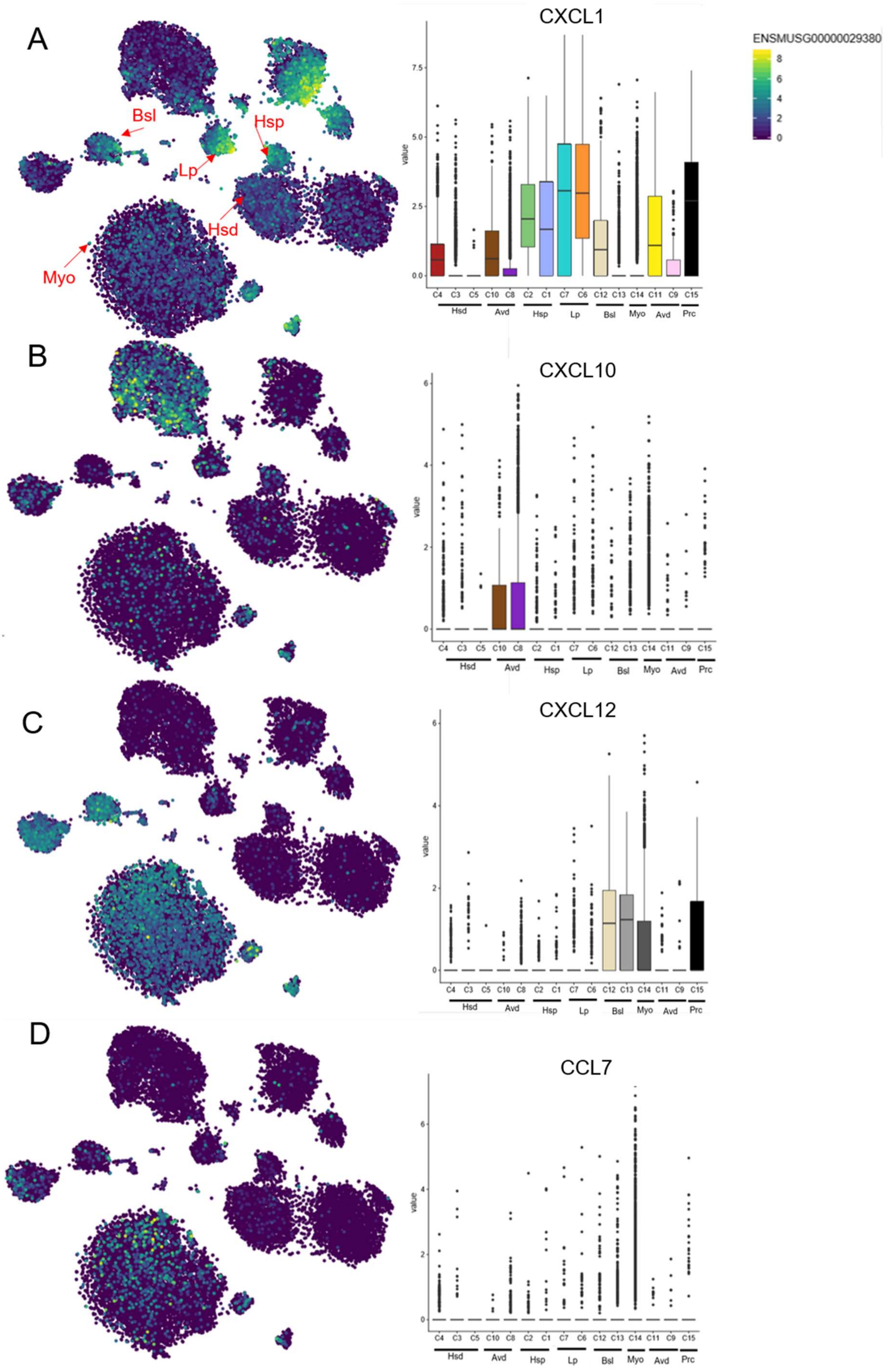
A
Bsl
Hsp
Lp
Hsd
Myo
CXCL1
ENSMUSG00000029380
value
C4 C3 C5 C10 C8 C2 C1 C7 C6 C12 C13 C14 C11 C9 C15
Hsd Avd Hsp Lp Bsl Myo Avd Prc
B
CXCL10
C
CXCL12
D
CCL7

Figure S4: Chemokines are produced by epithelial cell subsets.

Expression of **(A)** CXCL1, **(B)** CXCL10, **(C)** CXCL10 and **(D)** CCL7 by epithelial cells was determined by searching the single cell RNAseq data repository from Bach *et al*, 2017 (Bach *et al.*, 2017) at: https://marionilab.cruk.cam.ac.uk/mammaryGland/. Epithelial subsets include; hormone sensing differentiated (Hsd), differentiated alveolar (Avd), hormone sensing progenitor (Hsp), luminal progenitor (Lp), basal (Bsl), myoepithelium (Myo), Procr+ (Prc).

Development • Supplementary information

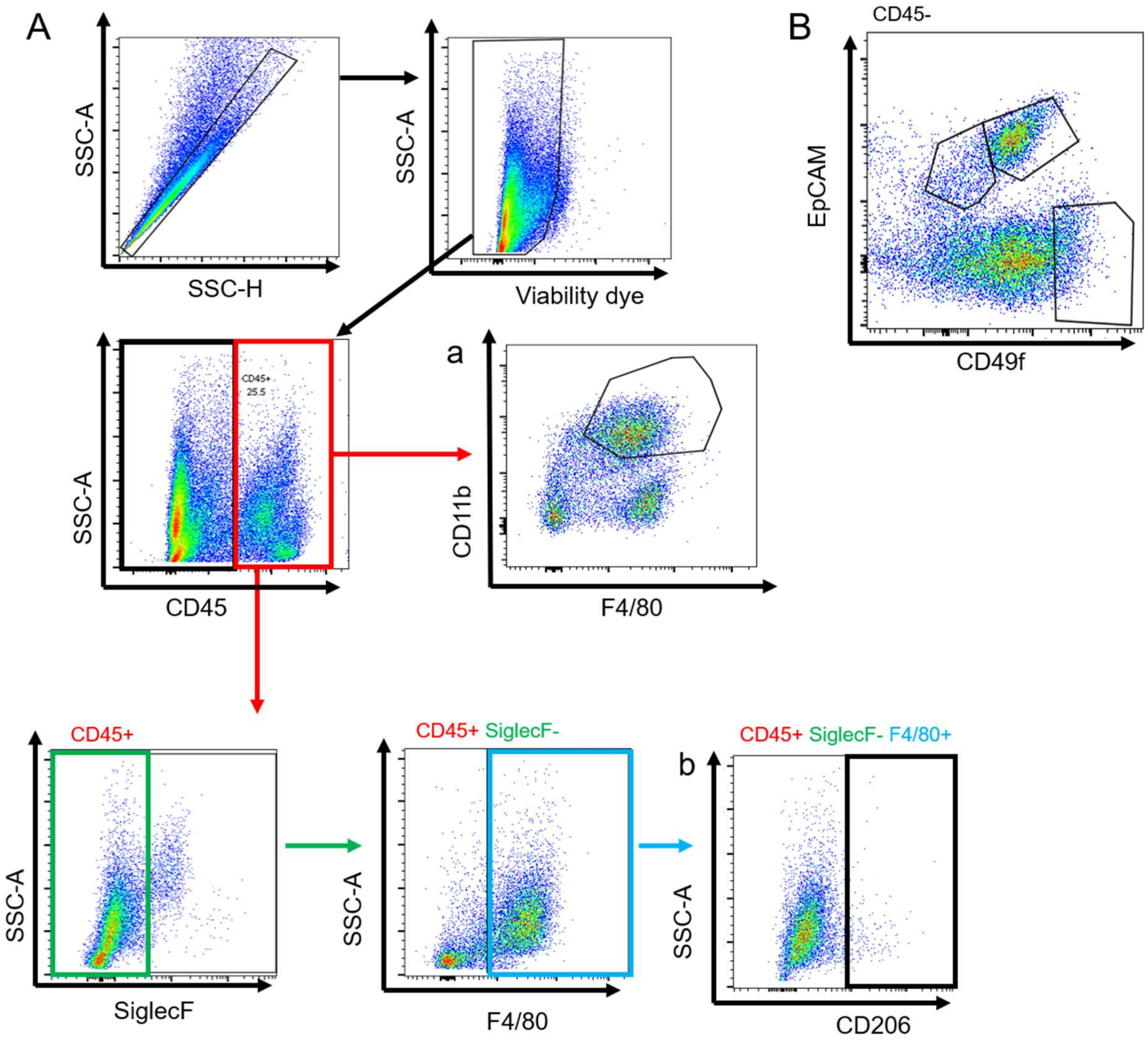

Figure S5: Gating strategy to define immune and epithelial cells in the mammary gland. Flow cytometry was carried out to measure the percentage of cells in the mammary gland. Initially, single cells were gated, dead cells were excluded, and CD45+ immune cells were gated. Populations were then expressed as a percentage of CD45+ cells, including; **(A) a)** CD11b+F4/80+, and **b)** SiglecF-F4/80+CD206+ cells. **(B)** For epithelial cell subsets, live, single CD45- cells were gated. Populations were then expressed as a percentage of CD45- cells, including mature (EpCAM+ CD49f-) and progenitor luminal (EpCAM+ CD49f+), and basal (EpCAM - CD49f+) cells.

Development • Supplementary information

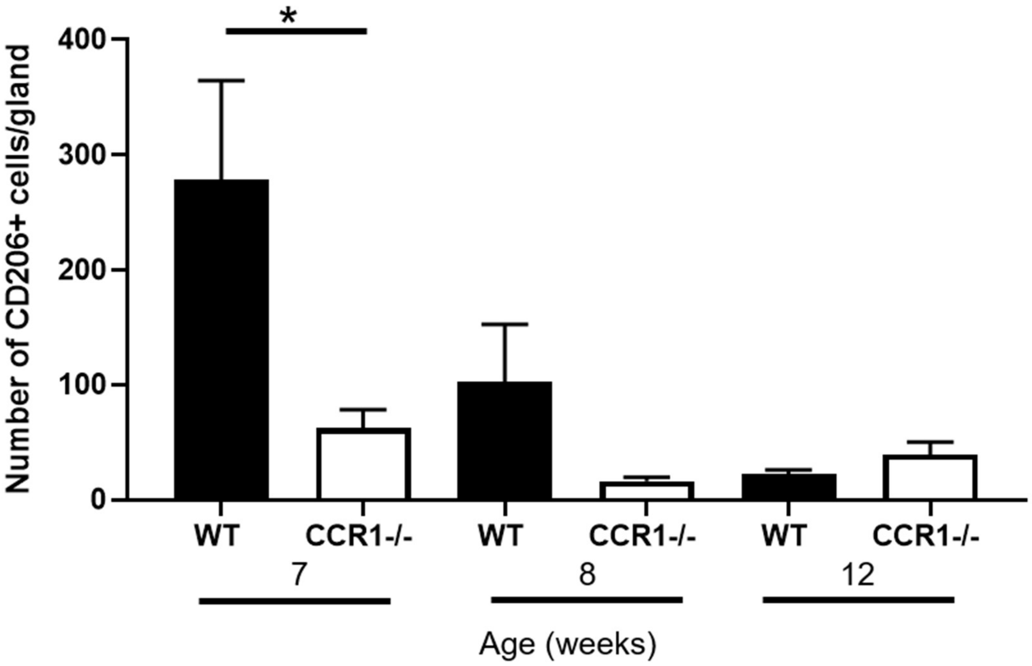

Figure S6: CD206+ macrophages throughout development. Flow cytometry was used to determine the number of SiglecF- F4/80+ CD206+ macrophages, within 7 (each group, n=6), 8 and 12 (WT, n=4 CCR1-/-, n=5) weeks old developing mammary glands. Significantly different results are indicated. Mann-Whitney test, $p=0.0152$. Error bars represent S.E.M.

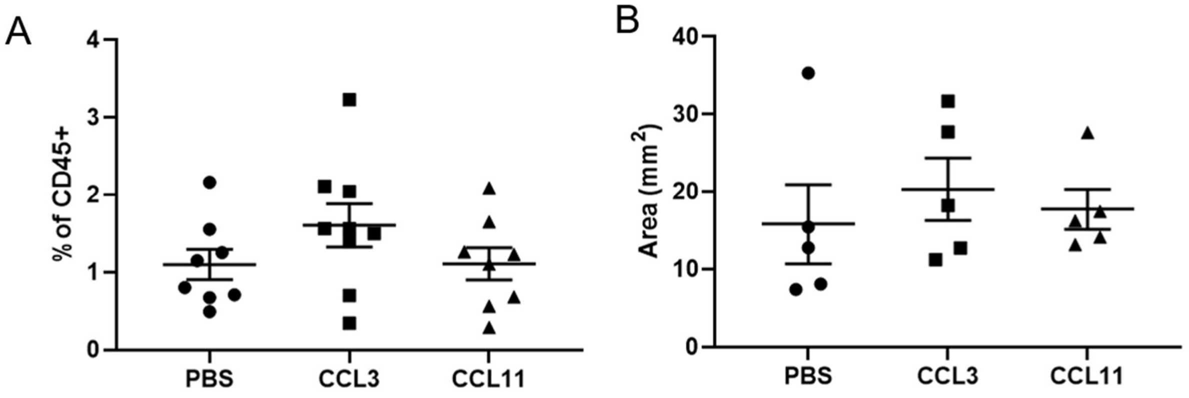

Figure S7: CCL3 and CCL11 do not control CD206+ macrophages or the extent of branching morphogenesis. 3 days after subcutaneous administration of PBS, 2 µg CCL3 or CCL11 at 6 weeks, (**A)** the percentage of SiglecF-F4/80+CD206+ cells measured by flow cytometry. (PBS, n=8, CCL11, n=8, CCL3, n=9) and (**B)** the area of branching was measured using Image J (each group, n=5).

Table S1: Pubertal onset in CCR1-/- mice

**Day***	**38**	**42**	**45**
**WT**	9/11 (81.8%)	11/11 (100%)	11/11 (100%)
**CCR1-/-**	5/8 (62.5%)	7/8 (87.5%)	8/8 (100%)

*Pubertal onset determined by assessing vaginal opening.

Development • Supplementary information
