# Supplementary material for: Chemokine receptors coordinately regulate macrophage dynamics and mammary gland development
Source: Development. 2020 Jun 17;147(12):dev187815. doi: 10.1242/dev.187815 (PMC7328164; doi:10.1242/dev.187815)
Supplement: Reviewer comments [file develop-147-187815_review_history.pdf]

# Chemokine receptors coordinately regulate macrophage dynamics and mammary gland development

Gillian J. Wilson, Ayumi Fukuoka, Samantha R. Love, Jiwon Kim, Marieke Pingen, Alan J. Hayes and Gerard J. Graham
DOI: 10.1242/dev.187815

**Editor**: Liz Robertson

**Review timeline**

| | |
|---|---|
| Original submission: | 30 December 2019 |
| Editorial decision: | 3 February 2020 |
| First revision received: | 20 April 2020 |
| Editorial decision: | 18 May 2020 |
| Second revision received: | 19 May 2020 |
| Accepted: | 20 May 2020 |

---

**Original submission**

First decision letter

MS ID#: DEVELOP/2019/187815

MS TITLE: Chemokine receptors coordinately regulate macrophage dynamics and mammary gland development

AUTHORS: Gillian J Wilson, Ayumi Fukuoka, Samantha R Love, Jiwon Kim, Alan Hayes, Marieke Pingen, and Gerard Graham

I have now received all the referees reports on the above manuscript, and have reached a decision. The referees' comments are appended below, or you can access them online: please go to BenchPress and click on the 'Manuscripts with Decisions' queue in the Author Area.

The overall evaluation is positive and we would like to publish a revised manuscript in Development, provided that the referees' comments can be satisfactorily addressed. Please attend to all of the reviewers' comments in your revised manuscript and detail them in your point-by-point response. If you do not agree with any of their criticisms or suggestions explain clearly why this is so.

Reviewer 1

*Advance summary and potential significance to field*

Wilson and colleagues investigated the CCR1/ACKR2 axis in mammary gland development. In a previous study the group showed that ACKR2 plays an important role in the branching of ductal epithelium. Here they extend the findings by pinpointing CCR1 as the inflammatory chemokine receptor the interplays with ACKR2 and suggest the chemokine CCL7 as the mediator in this process. While ACKR2 is expressed on stromal cells of the mammary gland, CCR1 is expressed on a specific CD206 positive / Siglec F negative subset of F4/80 macrophages.
The MS is well written and the conclusions plausible. The finding is new and elucidates a new aspect of CCR1 in development.

© 2020. Published by The Company of Biologists under the terms of the Creative Commons Attribution License (https://creativecommons.org/licenses/by/4.0/).

*Comments for the author*

However, some additional information/clarifications would improve the message of the MS.
1. Figure 4: the hormone estradiol increases CCR1 expression. It is unclear whether this is due to simple increased surface expression through relocation of CCR1 from intracellular compartments to the cell surface (1h incubation),
through enhanced translation or transcriptional activation and/or de novo synthesis. Some mechanistic data should be added.
2. Line 189 they report elevated chemokine levels in the absence of ACKR2. The results differ somewhat from a previous observations (Wilson 2017) and are in contradiction with the known ligands of ACKR2. What is the mechanism of the apparent CCL19, CXCL1, CXCL10 and CXCL12 upregulation? Given the variability of chemokine levels in the ACKR2ko replicas, four determinations may not be sufficient for the conclusions made.
3. Compared to other chemokines CCL7 and CCL3, both are expressed at low levels in mammary gland extracts and their concentration barely doubles in the absence of the scavenger ACKR2. On the other hand, injection of CCL7 appears to increase branching in WT animals. Does CCL3 (or additional chemokines) also induce the same branching? It would be important to analyze a potential role of CCL3 to underline the specificity of the CCL7/CCR/ACKR2 axis.

Minor points:
Lane 83 reference is missing.
Line 575/579 duplicate reference Line 619 incorrect citation Figure 2, 3, suppl. 2 x-axis tick marks and labeling should be improved.

<u>Reviewer 2</u>

*Advance summary and potential significance to field*

This paper shows that CCL7 regulates macrophage abundance via CCR1 during mammary gland development and as a consequence loss of this signalling pathway delays ductal development. This CCL7 action is also balanced during mammary development by its scavenging receptor ACKR2. To some extent this expression of CCR1 is regulated by estrogen that is required for mammary ductal development.

*Comments for the author*

The study is quite nicely done and the conclusions follow on from the data. These conclusions are interesting and extend the field. In some cases, the effect sizes are small but apparently significant.

There are a few things that would improve the paper:

1. The effect on mammary development in the CCR1 nulls is much smaller than that seen for complete loss of macrophages. This suggests that CCR1/CCL7 signalling is only part of the story. Can the authors speculate on this observation and also relevant to this point, did they explore the location of the macrophage in the chemokine nulls and also whether other structures such as collagen fibers are normal?

2. Figure 7 shows that eosinophils are the major source of CCL7. It has been reported that loss of eosinophils in the mammary gland reduces branching complexity (Gouon-Evans et al 2000). This data was not discussed but it would be revealing to compare phenotypes or indeed, to do the compound mutant experiment with a CCR1:CCL11 null.

3. There is rescue of the mammary gland branching defect in the mature mice. Have the authors confirmed that puberty (i.e. onset of estrus is normal in the CCR1 nulls)? Do they have a mechanism for this developmental recovery, are macrophages recruited through another mechanism late?

© 2020. Published by The Company of Biologists under the terms of the Creative Commons Attribution License (https://creativecommons.org/licenses/by/4.0/). 

4. There is an antibody problem for CCR1 with significant binding even in the CCR1 nulls (Fig 3a). Did the authors validate the data using PCR for example? Double staining of the RNA Scope with F4/80 would also improve the paper.

5. In Fig 6 what is F4/80 CD11b minus population?

6. Fig 4e not labelled.

7. Fig 1 – best to indicate CCL ligands on figure

---

**First revision**

<u>Author response to reviewers' comments</u>

**Response to Reviewers**

**DEVELOP/2019/187815**

**Reviewer 1 Advance Summary and Potential Significance to Field:**

**Wilson and colleagues investigated the CCR1/ACKR2 axis in mammary gland development. In a previous study the group showed that ACKR2 plays an important role in the branching of ductal epithelium. Here they extend the findings by pinpointing CCR1 as the inflammatory chemokine receptor the interplays with ACKR2 and suggest the chemokine CCL7 as the mediator in this process. While ACKR2 is expressed on stromal cells of the mammary gland, CCR1 is expressed on a specific CD206 positive / Siglec F negative subset of F4/80 macrophages. The MS is well written and the conclusions plausible. The finding is new and elucidates a new aspect of CCR1 in development.**

**Comments:**
**1. Figure 4: the hormone estradiol increases CCR1 expression. It is unclear whether this is due to simple increased surface expression through relocation of CCR1 from intracellular compartments to the cell surface (1h incubation), through enhanced translation or transcriptional activation and/or de novo synthesis. Some mechanistic data should be added.**

We agree with the reviewer that this is an important point and have added data (Fig. 4D) to show that transcription of CCR1 mRNA by purified CD11b+F4/80+ cells is increased in response to estradiol, suggesting that de novo synthesis rather than relocation of CCR1 is happening (lines 178-181).

**2. Line 189 they report elevated chemokine levels in the absence of ACKR2. The results differ somewhat from a previous observations (Wilson 2017) and are in contradiction with the known ligands of ACKR2. What is the mechanism of the apparent CCL19, CXCL1, CXCL10 and CXCL12 upregulation? Given the variability of chemokine levels in the ACKR2ko replicas, four determinations may not be sufficient for the conclusions made.**

We apologise for our unclear description of the proteomic data. We believe that the upregulation of chemokines which are not ACKR2 ligands, CCL19, CXCL1, CXCL10 and CXCL12, reflects either the altered immune cell content or the extent of epithelial cell branching, which is increased in ACKR2-/- and decreased in CCR1-/- glands. Indeed, bioinformatic analysis of mammary epithelial cell single cell data reveals that CXCL1, CXCL10 and CXCL12 are produced by epithelial cells. We have added this data to Supplementary Fig. 4 and have clarified this in the text (lines 201-207). In our previous study we used a more limited panel of luminex analytes which did not include CCL19, CXCL10 or CXCL12.

© 2020. Published by The Company of Biologists under the terms of the Creative Commons Attribution License (https://creativecommons.org/licenses/by/4.0/).

Although the ACKR2 sample size in this study was small, we were able to repeat our previous observations; elevated CCL7, CCL11 and CCL12 and unchanged CCL2 and CCL5. This study revealed additional ACKR2 ligands which are elevated, CCL3 and CCL22. We believe this may reflect the later time point of samples used for the Luminex in this study, 7 weeks, compared with the previous study which was carried out using samples from 6.5 weeks. This window of mammary gland development is characterised by rapid morphological changes.

**3. Compared to other chemokines CCL7 and CCL3, both are expressed at low levels in mammary gland extracts and their concentration barely doubles in the absence of the scavenger ACKR2. On the other hand, injection of CCL7 appears to increase branching in WT animals. Does CCL3 (or additional chemokines) also induce the same branching? It would be important to analyze a potential role of CCL3 to underline the specificity of the CCL7/CCR/ACKR2 axis.**

This is an excellent point and we have now included data showing that CCL3 and CCL11 do not increase the area of branching or the percentage of CD206+ macrophages (Lines 264- 268; Supplementary Fig. 7). Therefore, the CCL7/CCR1/ACKR2 axis appears to be specific.

**Minor points:**
**Lane 83 reference is missing.**

Thank you for pointing this out. We have included the correct reference.

**Line 575/579 duplicate reference.**

This error has been corrected.

**Line 619 incorrect citation**

This incomplete citation has been corrected.

**Figure 2, 3, suppl. 2 x-axis tick marks and labeling should be improved.**

We agree that these labels were not clear. They have been amended.

**Reviewer 2 Advance Summary and Potential Significance to Field:**

**This paper shows that CCL7 regulates macrophage abundance via CCR1 during mammary gland development and as a consequence loss of this signalling pathway delays ductal development. This CCL7 action is also balanced during mammary development by its scavenging receptor ACKR2. To some extent this expression of CCR1 is regulated by estrogen that is required for mammary ductal development.**

**Comments:**
**1. The effect on mammary development in the CCR1 nulls is much smaller than that seen for complete loss of macrophages. This suggests that CCR1/CCL7 signalling is only part of the story. Can the authors speculate on this observation and also relevant to this point, did they explore the location of the macrophage in the chemokine nulls and also whether other structures such as collagen fibers are normal?**

We agree that ACKR2 and CCR1 do not regulate all macrophage mediated effects on mammary gland development. In the absence of all four inflammatory chemokine receptors, CCR1, CCR2, CCR3 and CCR5, macrophages are markedly reduced in the mammary gland, but not depleted (For Reviewer Figure 1). We believe that macrophages which do not express iCCRs, such as those

© 2020. Published by The Company of Biologists under the terms of the Creative Commons Attribution License (https://creativecommons.org/licenses/by/4.0/).

recruited through alternative chemokine receptors such as CX3CR1 could also be important. We have added these details to the discussion (Lines 303- 307).
We agree that determining the location of macrophages and their effects on collagen deposition would be a nice addition to the manuscript and carried out staining to investigate this. Unfortunately, we were unable to complete these experiments before our lab was shut down due to COVID-19. If the reviewer feels this is essential, we would ask that we are given an extension until we are able to return to the lab.

**2. Figure 7 shows that eosinophils are the major source of CCL7. It has been reported that loss of eosinophils in the mammary gland reduces branching complexity (Gouon- Evans et al 2000). This data was not discussed but it would be revealing to compare phenotypes or indeed, to do the compound mutant experiment with a CCR1:CCL11 null.**
We apologise for not discussing this key data in the original manuscript and have added details to the discussion in Lines 308-314. As CCR3-/- mice also have reduced numbers of eosinophils, these mice can reveal insight into their contribution. The extent of branching is unaffected in CCR3-/- mice, suggesting that eosinophils do not directly control the extent of branching (Supplementary Fig. 1). In addition, injection of CCL11 into pubertal WT mice did not affect the branched area (Supplementary Fig. 7).

**3. There is rescue of the mammary gland branching defect in the mature mice. Have the authors confirmed that puberty (i.e. onset of estrus is normal in the CCR1 nulls)? Do they have a mechanism for this developmental recovery, are macrophages recruited through another mechanism late?**

This is an excellent point and we have now confirmed that the onset of puberty is normal in CCR1-/- mice by observing the day of vaginal opening (Table S1, Lines 123-4). To determine whether CD206+ macrophages are recruited later in CCR1-/- mice, we carried out flow cytometric analysis of the later time points of 7, 8 and 12 weeks (Supplementary Figure 6, lines 224-228). The number of CD206+ macrophages were not increased in CCR1-/- mice at any of the time points investigated. However, it is possible that these cells may be recruited in CCR1-/- mice at a time point between 8 and 12 weeks.

**4. There is an antibody problem for CCR1 with significant binding even in the CCR1 nulls (Fig 3a). Did the authors validate the data using PCR for example? Double staining of the RNA Scope with F4/80 would also improve the paper.**

We have validated these results using qRT-PCR. In our previous study we showed that CCR1 was expressed by F4/80+ cells using an RT2 PCR profiler array (Wilson et al, 2017). We have now further confirmed this by carrying out qRT-PCR to reveal CCR1 transcription by purified CD11b+F4/80+ cells (Fig 4D).
We agree that it would be good to include F4/80 double staining. Unfortunately, we were unable to complete this before the COVID-19 shut down.

**5. In Fig 6 what is F4/80 CD11b minus population?**

This CD11b-F4/80+ population appears to be a novel macrophage subtype in the mammary gland. These cells express MHCII and do not express CD11c, Ly6C, CD3, CD19, SiglecF or CD206. As these cells are not altered in the absence of CCR1, we have not discussed them in this manuscript (For Reviewer Figure 2). We would be happy to provide these details if the reviewer feels that they will improve the manuscript.

**6. Fig 4e not labelled.**

We apologise for our mistake. This has been corrected.

© 2020. Published by The Company of Biologists under the terms of the Creative Commons Attribution License (https://creativecommons.org/licenses/by/4.0/).

**7. Fig 1 - best to indicate CCL ligands on figure**

We agree with the reviewer and have amended Fig. 1.

---

Second decision letter

MS ID#: DEVELOP/2019/187815

MS TITLE: Chemokine receptors coordinately regulate macrophage dynamics and mammary gland development

AUTHORS: Gillian J Wilson, Ayumi Fukuoka, Samantha R Love, Jiwon Kim, Alan Hayes, Marieke Pingen, and Gerard Graham

I have now received all the referees reports on the above manuscript, and have reached a decision. The referees' comments are appended below, or you can access them online: please go to BenchPress and click on the 'Manuscripts with Decisions' queue in the Author Area.

The overall evaluation is positive and we would like to publish your manuscript in Development. One of the reviewers has suggested you cite a recent paper from the Visvader group. Could you please include this if appropriate and then re-upload the files. I will then accept the manuscript.

Reviewer 1

*Advance summary and potential significance to field*

The manuscript convincingly shows that ACKR2 and CCR1 fulfill opposite roles in the branching of breast epithelium.
The data of the revised MS support well the conclusions.

*Comments for the author*

No further comments.

Reviewer 2

*Advance summary and potential significance to field*

The new paper is improved with more detail. The phenotypes of the CCR1 knock out in mammary development are not great but appear real. There is also interesting new data added re regulation and cells expressing CCL7. I was sorry that some more detail could not be added because of the COVID situation, but I don't think this should hold up publication.

*Comments for the author*

The authors should add in the new reference published while this was under review from Visvader's group in Nature Cell Biology May 2020
"Tissue-resident ductal macrophages survey the mammary epithelium and facilitate tissue remodelling". This concludes in part that the macrophages are recruited from monocytes.

---

© 2020. Published by The Company of Biologists under the terms of the Creative Commons Attribution License (https://creativecommons.org/licenses/by/4.0/).

**Second revision**

Author response to reviewers' comments

The suggested citation has been included.

---

Third decision letter

MS ID#: DEVELOP/2019/187815

MS TITLE: Chemokine receptors coordinately regulate macrophage dynamics and mammary gland development

AUTHORS: Gillian J Wilson, Ayumi Fukuoka, Samantha R Love, Jiwon Kim, Alan Hayes, Marieke Pingen, and Gerard Graham
ARTICLE TYPE: Research Article

I am happy to tell you that your manuscript has been accepted for publication in Development, pending our standard ethics checks.

© 2020. Published by The Company of Biologists under the terms of the Creative Commons Attribution License (https://creativecommons.org/licenses/by/4.0/).
